# A comprehensive model for scaling up care for small and/or sick newborns at district level–based on country experiences presented at a WHO-UNICEF expert consultation

**DOI:** 10.7189/jogh.13.03023

**Published:** 2023-04-21

**Authors:** WHO-UNICEF Expert and Country Consultation on Small and/or Sick Newborn Care Group, Rajiv Bahl, Msandeni Chiume, Sumita Ghosh, Gagan Gupta, Patricia Fernandez, Muhammad Islam, Sartie Kenneh, Zeni Carvalho Lamy, Lily Kak, Hema Magge, Susan Niermeyer, Jesca Nsungwai, Yared Tadesse, Hoang Thi Tran, Teshome Desta Woldehanna

## IMPORTANCE OF SCALING UP SMALL AND/OR SICK NEWBORN CARE

Current trends indicate that 63 countries are not on track to achieve the 2030 Sustainable Development Goals (SDG) target of a neonatal mortality rate ≤12 per 1000 live births, with 55 needing to double the annual rate of decline in neonatal mortality to do so [1].

The global report on transforming care for small and/or sick newborns in 2019 called for urgent actions [2], articulating the importance of strengthening care for small and/or sick newborns at different levels of health facilities to accelerate progress. The Standards for Improving the Quality Care of Small and/or Sick Newborns in Health Facilities provide the framework, input, process, and outcome measures for such care [3]. The Every Newborn Action Plan (ENAP) 2020-25 sets a specific target for a functional special care unit for small and/or sick newborns in at least 80% of districts in each country [4]. Achieving this target will require a comprehensive model that describes what care is to be delivered and how countries and program managers can build and organize care delivery systems to scale up small and/or sick newborn care.

In the 2000s, there was a focus on scaling up basic preventative and promotive newborn care and services at community and first-level facilities, including hygiene, breastfeeding, maternal nutrition, and antenatal care [2]. This was rapidly followed by a scale-up of essential newborn care (immediate care at birth and resuscitation, thermal care, initiation of breastfeeding, prevention of infection, and recognition of danger signs). Additionally, there were efforts to scale up intrapartum care; about 80% of births now occur in a health facility, and optimizing the gains of this for newborn survival will require strengthening facility-based newborn care with strong community linkages. Special care for small and/or sick newborns in district-level hospitals represents the next frontier in the evolution of newborn care for accelerating the rate of reduction in neonatal mortality even without access to neonatal intensive care [5,6]. Historically, neonatal mortality rates of 12 per 1000 live births were achieved in the United Kingdom and United States only after the introduction of hospital-based special newborn care, and single-digit neonatal mortality was associated with widespread access to neonatal intensive care [7]. More recent examples provide additional support for this approach [8]. While most low- and middle-income countries (LMICs) are in the early stages of this journey, **Table 1** shows examples of successful scale-ups despite resource limitations. The experience of such countries, including factors enabling success and gaps identified in their contexts, can inform an adaptable model for countries in the planning stage. Here we describe such a model to consolidate learning to date and accelerate progress globally.

**Table 1:** Key lessons from countries scaling up small and/or sick newborn care

Key achievements	Factors enabling success	Gaps in implementation
**India:** SNCU established in 85% of districts as part of comprehensive system of facility-based and home-based newborn care including referral transport and follow-up at home and facility levels.	Strong government leadership, ownership by states and national plan for scale up. Adequate domestic resources allocation with clearly defined budget lines in district, state, and national plans. Government investment in additional human resources for SNCUs. Development of operational guide and tool kit for standardization of design and services for program managers. Partnership with UNICEF and National Neonatology Forum for implementation and training. Strong data systems. Adaptive learning and evolution over time (eg, introduction of follow up system with community-facility linkages, district early intervention clinics, family-centred care.	Need to improve linkages with maternal health care, particularly intrapartum care. Need to develop midwifery and neonatal nursing cadres. Need to increase coverage of key interventions such as KMC and CPAP.
**Bangladesh:** SCANUs established in 50 of 64 districts (78%) on well-developed foundation of primary care.	Strong commitment of government and development partners. National Newborn Health Program and national newborn working team. Engagement of professional bodies and academia. Development of a center of excellence for newborn care. Data system for tracking, monitoring, quality improvement. Partnership with UNICEF for implementation support.	Need to improve referral and follow-up system. Need to improve availability and capacity of human resources for SCANU. Need to strengthen system for equipment maintenance. Low community engagement. Gaps in domestic financing for scale-up with partial dependency on partners funding.
**Malawi:** 54 Newborn Care Units established in 28 districts with focus on multi-disciplinary capacity-building for special newborn care and effective maintenance and use of equipment.	Government leadership with inclusion of neonate in national health agenda; newborn steering committee. Collaborative implementation with government, development partners, NEST 360, educational institutions, professional associations.	Inadequate health financing. Insufficient trained health care workers. Limited space for newborn care. Inadequate, erratic supply of medicines, equipment, and commodities. Low community engagement.
**Sierra Leone:** Establishment of SBCUs in 14 district hospitals nationwide and scaling up KMC into small and/or sick newborn care services.	Advocacy by the First Lady to establish a SNCU in every district hospital. Leadership and ownership by government with partner funding and support from UNICEF. National standard package for scale-up of SCBUs. Quality management networking with engagement of health facility administration. Strong investment in equipment maintenance and building local capacity on same.	Sustainability of SCBUs without outside funding. Insufficient human resources. Lack of electricity and ancillary services in some facilities. Weak data system.
**Vietnam:** SNCU in most district level health facilities with interventions focused on integration of immediate and continuing KMC into small and/or sick newborn care.	National Action Plan on Maternal Newborn and Child Health. Policy and coverage targets for Early Essential Newborn Care and KMC. Monitoring framework and review process.	Limited equipment and commodities for small and sick newborn care. Regional disparities in care. Limited monitoring and technical support. Gap in functional network of care between national/regional and district hospitals.
**Argentina:** SNCU in most district level health facilities in setting of national legal framework and regulations in support of newborns and system for post-discharge follow-up of at-risk newborns.	Strong national legal and operational frameworks. Regionalized perinatal care. Online perinatal information system. In-person and telehealth training structure for multidisciplinary teams.	Integration of inter-sectoral care (health, education, social welfare). Limited community engagement.
**Brazil:** Reference maternity hospitals in all 27 national federal units for KMC and a line of care for small and/or sick newborns, from identification of risks during pregnancy to neonatal hospitalization and follow-up after discharge in partnership with primary.	Organization of support structures for the implementation of the line of care. Online indicator monitoring system. Annual evaluation as feedback and to develop action plans. Strong structure for in-person and distance training for multidisciplinary teams.	Limited articulation between specialized care and primary care. Coverage for training of primary care teams.

SNCU – special newborn care units, KMC – kangaroo mother care, CPAP – continuous positive airway pressure, SCANU – special care newborn units, SBCU – special baby care units

## PROCESS OF DEVELOPMENT OF A MODEL FOR SMALL AND/OR SICK NEWBORN CARE

### WHO-UNICEF Expert and Country Consultation on Small and/or Sick Newborn Care Group

To initiate the development of a model of small and/or sick newborn care, WHO and UNICEF organized a global consultation in December 2021. The meeting aimed to share country experiences in scaling up inpatient newborn care units for small and/or sick newborns, to build consensus on key elements of a model for scale-up that can be adapted to local contexts, and to discuss the development of a draft outline for implementation guidance. Approximately 500 participants from more than 43 countries (focused on representation from ENAP priority countries) attended the three-day online consultation. Representatives from ministries of health in nine countries (South-East Asia, Western Pacific, sub-Saharan Africa, and Latin America) summarized their experiences in implementing special newborn care at secondary-level facilities. Invited specialists synthesized journeys and pragmatic aspects of scale-up and presented relevant new research on immediate kangaroo mother care (KMC) [9], guidance from WHO on human resource strategies to improve newborn care in LMICs [10], and innovative advances in technology in care for small and/or sick newborns, lactation management and human milk banking, maternal well-being and mental health, and infant- and family-centred developmental care [11,12]. These presentations generated active discussion facilitated by simultaneous translation in Arabic, English, French, Portuguese, and Spanish and the capture of comments in the online chat. Break-out groups provided input into the model’s key elements, which are summarized here.

**Figure:**
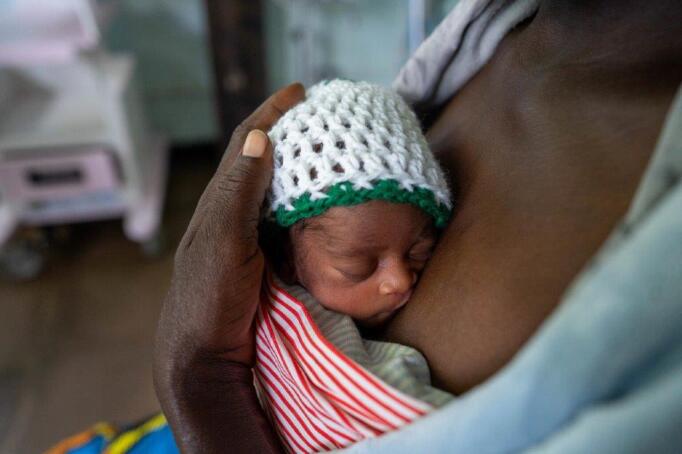
Photo: Portrait of baby Tamara wrapped safe in her mother’s Kangaroo pouch. She was under the care of the Neonatal Intensive Care Unit at Nebbi General Hospital after being born weighing 1.3kg. UNICEF and partners are supporting newborn care initiatives at Nebbi General Hospital including provision of equipment to keep small babies warm. Source: UNICEF WeShare. Available: https://weshare.unicef.org/archive/-2AMZIF69WQD9.html. © UNICEF/Zahara Abdul.

The most commonly cited success factors included strong national leadership committed to newborn health and a national newborn steering group with a long-term vision. Adequate financing was often achieved through a combination of government funds, investment of development partners, and UN agency resources. Implementation of scale-up depended upon collaboration of governments, academic institutions, professional associations, and development partners. Developing standardized approaches to scale-up and investing in human resources (both structure and capacity) and strong data systems favoured success.

Countries identified additional factors that may need to be addressed as implementation advances. Leadership and the ability to execute the plan from national to district implementation site are key to implementation success. Sustainable financing that utilizes domestic resources to support special newborn care is necessary to preserve quality and avoid loss of initial investment. Strengthened data systems, functional referral systems within networks of care, and specialized health care workers often grow as systems of special newborn care mature. Countries already advanced in scale-up and those just beginning both highlighted challenges with engaging families and communities to reduce inequities. Most countries learned by doing, adopting, and adding newer components and missing pieces based on emerging needs as they moved ahead. We believe that the learning curve of five to 10 years can be shortened for countries that are embarking on their journey by harvesting this learning to identify critical components for a scalable model for small and/or sick newborn care.

## MODEL FOR SCALE-UP OF SMALL AND/OR SICK NEWBORN CARE

**Figure 1:**
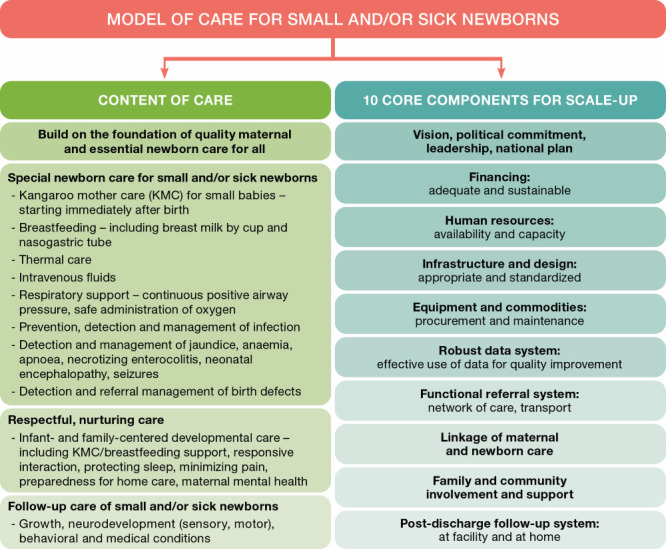
A model of small and/or sick newborn care: What care is required and how to scale up.

The model of small and/or sick newborn care incorporates both the content of care (what care should be provided to small and/or sick newborns) and 10 core components for scaling up care for small and/or sick newborn care at the district level (how to scale up) (**Figure 1**). The model builds upon a strong foundation of quality maternal care and essential newborn care [13]. While emphasizing the fundamentals of periconceptional, antenatal, and intrapartum care, special newborn care also implies coordinated delivery of maternal interventions that are primarily directed at the health of the foetus/newborn, such as antenatal corticosteroids for threatened preterm birth and timely caesarean-section delivery for maternal or fatal indications. Similarly, the content of newborn care recognizes the lifesaving importance of essential interventions including skin-to-skin contact to avoid hypothermia, facilitate bonding, and reduce stress and pain as well as early initiation of exclusive breastfeeding, delayed umbilical cord clamping, and resuscitation when needed; however, the actions to deliver these essentials might be modified when caring for small and/or sick newborns [3].

### The content of small and/or sick newborn care

The content of care for small and/or sick newborns addresses both provision and experience of care, as delineated in the WHO quality of care framework [14]. Evidence-based interventions for the provision of special newborn care correspond to those outlined for second-level care in the Survive and Thrive: transforming care for every small and/or sick newborn [2]. Experience of care for newborns in health facilities depends fundamentally on involvement of the family (zero separation) in infant- and family-centred developmental care [12,15]. After discharge, besides the care provided by parents and primary care professionals, specialized follow-up is necessary to address continued medical and developmental needs [16].

The care for small and/or sick newborns must build on quality maternal care and essential newborn care for all mothers and newborns. This is critical not only for the prevention of complications, but also for improving outcomes of small and/or sick newborns.

The first aspect of the content of small and/or sick newborn care in second-level newborn care units is that it incorporates supportive care (thermal, nutritional, and respiratory support), as well as prevention, recognition, and management of specific conditions associated with prematurity, asphyxia, and infection (**Figure 1**).

The second aspect is that small and/or sick newborns should receive respectful and nurturing care. KMC should anchor care for small newborns, as it provides high-quality thermal protection, promotes breastfeeding/feeding of breast milk, prevents infection, empowers the mother to care for her baby, and supports the reciprocal interaction between newborn and parent that underlies nurturing neurodevelopmental care and integration of the family unit [17,18]. Immediate KMC for all small babies requires that newborn care units organize specialized care for mother and baby together immediately after birth. Direct involvement of families in the delivery of care opens opportunities for emotional and social support during hospitalization and leads to better preparedness for discharge.

The third aspect of special newborn care extends beyond discharge to provide systematic follow-up to all small and/or sick newborns with respect to growth, sensory function (hearing, vision), cognitive and motor development, and behavioural and medical conditions.

### Ten core components to scale up small and/or sick newborn care

Scaling up the model for care of small and/or sick newborn includes 10 key components that need to be developed simultaneously rather than sequentially. These are well aligned with the WHO health system building blocks and quality of care standards [4].

#### Component 1: Vision, political commitment and leadership, national plan

Central to the success of scale-up is the long-term vision, political commitment, and government leadership at all levels with a well-defined national scale-up plan. The national plan should clearly define the content of care at different levels of health facilities, including the critical components and resources needed for scale-up. Most countries that have embarked on second-level care have done so with the guidance of a national technical advisory committee responsible for situational assessment, setting achievable goals and timelines, and contextualizing guidance to the country situation.

#### Component 2: Financing

Leadership and political commitment must be accompanied by financial commitment reflected in national, provincial/state, and district budgets. While sources and amounts of funds vary (domestic or mix of donor and domestic), they adequately fit the defined scope and cover not only one-time development costs, but also running costs of maintenance, consumables, equipment replacement, and personnel. Sustainable health financing also requires country capacity to mobilize and effectively manage resources to support and further develop priority interventions around special newborn care while demonstrating accountability and achievement of quality results. Further, strategies for financial protection for families of small and/or sick newborns should be in place.

#### Component 3: Human resource

A sufficient number of health care workers 24/7, particularly nurses, is crucial to providing quality inpatient newborn care. Healthcare workers must have specialized competencies to care for small and/or sick newborns and effectively incorporate the family into caregiving in the facility. An interprofessional team comprised of both clinical and support staff, with mechanisms for ongoing mentorship and supportive supervision, continuity and non-rotation of trained staff, and attention to stress and burnout of staff, is critical to sustaining quality and fostering motivation.

#### Component 4: Infrastructure and design

Infrastructure and floor plans should include space for provisioning all needed services, including comfortable seating for mothers beside their infants to facilitate infant feeding and skin-to-skin contact. Shared facilities (including toilets, bathing rooms, food preparation area, follow-up clinic, counselling room, and triage) should be designed with consideration for local cultural norms around privacy and community traditions, and incorporated into national standards for specific hospital designs. Bed capacity should be determined based on projected population coverage needs, the number of inborn and outborn deliveries and referrals with room for growth. Uninterrupted power supply, adequate water, sanitation, and hygiene along with measures for infection prevention and control and safe waste disposal are minimum requirements. Innovations in architectural design to meet the needs of modern woman- and baby-centred care are needed to find cost-effective ways to facilitate care for mothers and babies together and support necessary companionship through labour, birth, and postnatal/neonatal intensive care unit (NICU) care.

#### Component 5: Equipment and commodities (including medicines)

Equipment procurement can be either done by governments or supported by partners. In both cases, it is important to have a standardized list and specifications of equipment with a clear plan for installation, training, and maintenance and building national capacity for the same. There should be adequate provisions for timely replenishment of consumables and medicines to avoid stockouts. Equipment must be robust to function in the face of variable power supply and dusty environments, and local systems for routine maintenance, repair, and replacement by bioengineers and technicians are essential to uninterrupted provision of care.

#### Component 6: Robust data system

Whether paper-based, digital, or a combination, facility data systems are vital not only for providing clinical care and monitoring outcomes, but also for tracking the quality of care, prioritizing mentorship and supportive supervision, and supporting advocacy and decision-making. Local data collection and use are fundamental to identifying problems early, triggering corrective actions, and improving the quality of care.

#### Component 7: Functional referral system

A network of care assures that patient needs are met at a facility that provides appropriate services and that health facilities are not overburdened with care that could be provided at a lower level or closer to a patient’s home. Second-level newborn care does not exist in isolation; recognition of emergent conditions that require advanced care during pregnancy or after birth demands efficient and purposeful referral from primary care sites to a facility that can provide that care. Where intensive or subspeciality care is available, referral from second- to third-level facilities requires coordinated dispatch and communications as well as trained and equipped transport personnel.

#### Component 8: Linkage of maternal and newborn care

Linkage of maternal and newborn care at every health system level and at every phase of care, from antenatal through intrapartum to postnatal, is key to providing preventive care, avoiding separation of mother and newborn at the time of referral, and achieving the goal of zero separation during in-facility care. Early recognition of risk factors helps direct patients to an appropriate facility for delivery. Intrapartum monitoring and care for the newborn in the delivery area or operating theatre are essential to reducing asphyxia and hypothermia, an avoidable mortality risk. Provision of care to mother and baby together in mother-newborn units, even when the baby requires respiratory support, has been shown to improve survival and reduce infection [9].

#### Component 9: Family and community involvement and support

Involvement of family members in small and/or sick newborn care will require policy changes in many systems; however, involvement of parents in the care for their newborn improves developmental outcomes, can improve the well-being of families and health care workers, and prepares the family to assume full care of their infant after discharge. Increasing community engagement calls for incorporating input from families and community leaders in the planning and evaluation of health services.

#### Component 10: Post-discharge follow-up system

A system of follow-up care after facility discharge is necessary to continue meeting the special needs of babies born small and/or sick. Optimal quality of survival depends on continued care at home and outpatient follow-up, not only to monitor the growth of small babies and medical conditions of those who were sick, but also to monitor and intervene for conditions that affect sensory function (hearing and vision), cognitive and motor development, and behavioural health. These specialized services also demand interprofessional teams and systems that connect smoothly from inpatient care. The protocol for follow-up after discharge at home/community and in a health facility should be well defined both in schedule and content of follow-up visits.

## CONCLUSION AND FUTURE DIRECTIONS

The model for scaling up small and/or sick newborn care represents a first step in support for countries implementing quality standards and achieving the 2025 ENAP target for small and/or sick newborn care. This model is intended to serve as the framework around which to build a learning community that adapts, tests, and adjusts the model based on local context and contributes to global learning. Promoting and supporting research on health system innovations for small and/ or sick newborn care will further enhance related coverage and quality of care. The global community must work together to achieve the collective goal and vision to end preventable newborn deaths and help newborns thrive.

18Ministry of Health Brazil. [Humanized Care for the Newborn Kangaroo Method]. 2017. Available: http://bvsms.saude.gov.br/bvs/publicacoes/atencao_humanizada_metodo_canguru_manual_3ed.pdf. Accessed: 14 March 2023. [in Brasilian].
